# Comparison of a New Optical Biometer That Combines Scheimpflug Imaging With Partial Coherence Interferometry With That of an Optical Biometer Based on Swept-Source Optical Coherence Tomography and Placido-Disk Topography

**DOI:** 10.3389/fmed.2021.814519

**Published:** 2022-02-10

**Authors:** Shihao Chen, Qiaoyue Zhang, Giacomo Savini, Shuangzhe Zhang, Xiaomin Huang, Jinjin Yu, Yirang Wang, Rui Ning, Jinhai Huang, Ruixue Tu

**Affiliations:** ^1^Eye Hospital and School of Ophthalmology and Optometry, Wenzhou Medical University; State Key Laboratory of Optometry, Ophthalmology and Vision Science, Wenzhou, China; ^2^Eye Institute and Department of Ophthalmology, Eye & ENT Hospital, Fudan University; Key Laboratory of Myopia, Chinese Academy of Medical Sciences, Shanghai, China; ^3^Department of Ophthalmology, Air Force Medical Center, Beijing, China; ^4^IRCCS G.B. Bietti Foundation, Rome, Italy; ^5^Shanghai Research Center of Ophthalmology and Optometry, Shanghai, China

**Keywords:** Scheimpflug imaging, swept source optical coherence tomography, repeatability, reproducibility, agreement

## Abstract

**Purpose:**

To evaluate measurement precision and to compare the Pentacam AXL (Oculus Optikgeräte, Wetzlar, German), a new optical biometer based on Scheimpflug imaging and partial coherence interferometry (PCI) with that of the OA-2000 biometer (Tomey, Nagoya, Japan), which combines swept-source optical coherence tomography (SS-OCT) and Placido-disk topography.

**Methods:**

Axial length (AL), central corneal thickness (CCT), anterior chamber depth (ACD), aqueous depth (AQD), mean keratometry (Km), astigmatism vectors J0, J45, and corneal diameter (CD) were measured in triplicate by two technical operators. Within-subject standard deviation (Sw), repeatability and reproducibility (2.77 Sw), coefficient of variation (CoV), and intraclass correlation coefficient (ICC) were used to assess the Pentacam AXL intra-observer repeatability and inter-observer reproducibility. Paired *t*-test and Bland-Altman plots were used to determine the agreement between the two biometers.

**Results:**

The new optical biometer had high intra-observer repeatability [all parameters evaluated had low CoV (<0.71%) and high ICC (>0.88)]. Inter-observer reproducibility was also excellent, with high ICC (>0.95) and low CoV (<0.52%). The 95% LoA between the new biometer and OA-2000 were insignificant for most of the parameters evaluated, especially for AL. However, the measurement agreement was moderate for CCT.

**Conclusions:**

Intra-observer repeatability and inter-observer reproducibility were excellent for all parameters evaluated using the new optical biometer based on Scheimpflug imaging and PCI. There was a high agreement between the two devices and hence could be clinically interchangeable for the measurement of most ocular parameters.

## Introduction

Accurate measurements of ocular biometric parameters are crucial for both intraocular lens (IOL) power calculation and refractive surgery (1–3). Over the last 20 years, optical biometry has become the gold standard to measure parameters such as axial length (AL) and chamber depth (ACD) (4–6). Recently, a new optical biometer (Pentacam AXL, Oculus, Germany), which combines Scheimpflug imaging and partial coherence interferometry (PCI) has been introduced into the market. AL measurements using the new biometer is based on PCI, whereas measurement of anterior segment parameters, such as corneal curvature and thickness, relies on a rotating Scheimpflug high-definition camera. Although the Pentacam (without PCI) has been widely used in ophthalmology, the measurement accuracy of the new optical biometer has yet to be determined. Recent studies have demonstrated that the OA-2000 (Tomey, Japan), an optical biometer that combines swept-source optical coherence tomography (SS-OCT) with Placido disc topography, offers good repeatability and reproducibility for measuring biometrical parameters. In addition, OA-2000 has been demonstrated to have a high agreement with other biometers in the market for most of the ocular parameters evaluated (7, 8). However, only a few studies have comprehensively investigated the precision of this new biometer based on the Bland-Altman criterion (9). Our initial aim of this study was to prospectively evaluate the intra-observer repeatability and inter-observer reproducibility of the measurements obtained using the Pentacam AXL. Our second aim was to compare the measurement agreement between these two optical biometers.

## Materials and Methods

### Subjects

This prospective observational study included 133 consecutive subjects from the Eye Hospital of Wenzhou Medical University, Wenzhou, China. Healthy subjects and patients with cataracts were enrolled in this study. All procedures adhered to the Declaration of Helsinki, and the study protocol was reviewed and approved by the Research Review Board of Wenzhou Medical University. The inclusion criteria were as follows: age ≥18 years, good fixation, patients had not worn rigid and soft contact lenses for at least 4 and 2 weeks, respectively. Subjects had intraocular pressure between 10 and 21 mmHg, absence of eye diseases except for refractive errors and cataracts. Subject exclusion criteria were as follows: a history of ocular surgery or trauma, active ocular pathology, and systemic diseases affecting the eyes.

### Instruments

The Pentacam AXL (Oculus, Germany) (software version 1.20r134)combines Scheimpflug imaging and PCI. It uses a blue light-emitting diode (LED) with a wavelength of 475 nm as the light source and a rotating Scheimpflug camera (180 degrees) that provides a 3-dimensional scan of the eye. It captures 25 images to obtain 138, 000 true elevation points from the front and back of the cornea surface (10, 11). From these data, curvature and thickness of the cornea are obtained. For this study, several corneal power values obtained from the Scheimpflug camera and mean keratometry (K) calculated using the 1.3375 keratometric index were considered. In addition, the Scheimpflug camera is able to measure the anterior chamber depth (ACD, from epithelium to the lens), aqueous depth (AQD, from the endothelium to the lens) and CD (corneal diameter). AL is measured using PCI which has a laser diode that emits 780 nm near-infrared short-coherent light (coherent length of approximately 160 μm).

The OA-2000 (software version 3C) is based on the principles of SS-OCT and Placido disk topography. The topographer has nine rings with 256 points projecting onto the cornea in a 5.5 mm zone. It is used to measure corneal parameters, such as K over a 2.5 mm and 3.0 mm diameter (the latter was investigated in this study). The SS-OCT was designed for measuring AL, central corneal thickness (CCT), ACD and lens thickness. The SS-OCT light source is a swept-source laser set at a wavelength of 1,060 nm. It can effectively reduce scattering and attenuation from the penetrating tissue (8, 12).

### Procedures

For each subject, only one randomly selected eye was measured. Measurements were performed randomly using the two biometers. Each eye was evaluated on the same day using the two biometers. All measurements were performed between 10:00 am to 17:00 pm in order to reduce the effect of diurnal variation, and all measurements were acquired within a time period of 30 min (13, 14).

The subject was asked to sit in front of the biometer, keep both eyes open and focus on a target. The subject was asked to blink before each scan was performed. Two experienced technicians scanned each eye three times using both the biometers. The order of both biometers and experienced technicians were randomized. Scans with a quality specification of “OK” (for the Pentacam AXL) and scans without a red mark (for the OA-2000) were considered for analysis. For intra-observer repeatability, all eyes were scanned using each biometer in triplicate by the same technician. For inter-observer reproducibility, the same measurements were repeated using the same new biometer by the other technician.

AL, ACD, AQD, CCT, mean K over 3.0 mm diameter and corneal diameter (CD) were recorded. Astigmatism was analyzed using the J0 and J45 vectors and calculated according to the following formulas (15).


(1) $$\begin{array}{r} \text{J}0=-{\left(C/2\right)}^{*}\cos\left(2^{*}A\right) \end{array}$$

(2) $$\begin{array}{r} \text{J}45=-{\left(C/2\right)}^{*}\sin\left(2^{*}A\right) \end{array}$$

(C = cylinder, A = axis).

### Statistical Analysis

Statistical analysis was performed using SPSS (version 21.0, IBM Corporation, USA), and results were presented as mean ± standard deviation (SD). A *p* < 0.05 was considered statistically significant. The Kolmogorov-Smirnov test was used to evaluate normal distribution of data (*P* > 0.05). To evaluate intra-observer repeatability and inter-observer reproducibility of the Pentacam AXL, the within-subject SD (Sw), test-retest repeatability (TRT), coefficients of variation (CoV) and intraclass correlation coefficient (ICC) were calculated. TRT, defined as 2.77Sw, shows the interval within which 95% of the differences between the measurements are expected to lie (16). The lower the TRT value and CoV, the better is the repeatability. ICC values ranged between 0 and 1. ICC values >0.75 denotes good repeatability, while values >0.9 suggests high repeatability (17). Agreement between the Pentacam AXL and OA-2000 were evaluated using paired *t*-test and Bland-Altman plots with 95% limits of agreement (LoA), which was defined as the mean difference ± 1.96 SD of difference (18).

## Results

This study enrolled 133 eyes of 133 subjects (71 females), including 69 healthy subjects and 64 patients with cataracts, with a mean age of 42.95 ± 19.95 years (range 23–63 years).

### Intra-observer Repeatability

The repeatability outcomes of measurements using the biometer based on Scheimpflug imaging and PCI are shown in Table 1. For the first observer, the intra-observer repeatability of the new biometer was high for all parameters, i.e., the CoV of AL, CCT, AQD, ACD, Km (3.0 mm) and CD were lower than 0.71%. For J0, J45, and Km, the TRT was no >0.3 D. The TRT was lower than 10.5 μm for CCT and lower than 0.2 mm for AL, AQD, ACD, and CD. The ICC for all parameters was higher than 0.88. In addition, the intra-observer repeatability was high for the second observer and was even better compared to the first observer. The CoV for all parameters were lower than 0.65%. Taking all the ocular parameters into account, the AL measurements had the best outcomes for both observers.

**Table 1:** Intraobserver repeatability outcomes for biometric measurements obtained using the new Scheimpflug imager in combination with partial coherence interferometry biometer.

**Parameter**	**Observer**	**Mean ±SD**	**S_**w**_**	**TRT**	**CoV (%)**	**ICC (95% CI)**
AL (mm)	1st	24.37 ± 1.49	0.04	0.11	0.16	0.999 (0.999–0.999)
	2nd	24.38 ± 1.49	0.04	0.10	0.15	0.999 (0.999–1.000)
CCT (μm)	1st	535.35 ± 29.68	3.73	10.33	0.70	0.984 (0.979–0.988)
	2nd	535.04 ± 29.56	3.49	9.67	0.65	0.986 (0.982–0.990)
AQD (mm)	1st	2.87 ± 0.45	0.02	0.05	0.68	0.998 (0.997–0.999)
	2nd	2.86 ± 0.45	0.02	0.05	0.61	0.999 (0.998–0.999)
ACD (mm)	1st	3.40 ± 0.45	0.02	0.06	0.62	0.998 (0.997–0.998)
	2nd	3.40 ± 0.45	0.02	0.05	0.56	0.998 (0.998–0.999)
Km (D)	1st	43.70 ± 1.55	0.10	0.28	0.23	0.996 (0.994–0.997)
	2nd	43.70 ± 1.56	0.10	0.27	0.22	0.996 (0.995–0.997)
J_0_ (D)	1st	−0.26 ± 0.42	0.06	0.17	−23.16	0.979 (0.973–0.985)
	2nd	−0.25 ± 0.42	0.06	0.17	−23.97	0.979 (0.972–0.985)
J_45_ (D)	1st	0.00 ± 0.21	0.07	0.19	−1,417.77	0.896 (0.864–0.922)
	2nd	0.00 ± 0.20	0.07	0.19	1,490.90	0.894 (0.862–0.921)
CD (mm)	1st	11.53 ± 0.39	0.06	0.18	0.56	0.974 (0.965–0.980)
	2nd	11.54 ± 0.38	0.06	0.17	0.55	0.974 (0.965–0.980)

*AL, axial length; CCT, central corneal thickness; AQD, aqueous depth; ACD, anterior chamber depth; Km, mean keratometry; CD, corneal diameter; SD, standard deviation; S_w_, within-subject standard deviation; TRT, test-retest repeatability (2.77 S_w_); CoV, within-subject coefficient of variation; ICC, intraclass correlation coefficient*.

### Inter-observer Reproducibility

The inter-observer reproducibility outcomes of the Scheimpflug/PCI based-biometer are shown in Table 2. The CoV of the ocular parameters were lower than 0.52%. In addition, the TRT of J0 and J45 were lower than 0.12D. The ICC for all the ocular parameters were higher than 0.9. These measurement outcomes demonstrated that inter-observer reproducibility for the above-mentioned parameters was high.

**Table 2:** Interobserver reproducibility outcomes for biometric measurements obtained using the new Scheimpflug imager in combination with partial coherence interferometry biometer.

**Parameter**	**Mean ±SD**	**S_**w**_**	**TRT**	**CoV (%)**	**ICC (95% CI)**
AL (mm)	24.37 ± 1.49	0.02	0.06	0.09	1.000 (1.000–1.000)
CCT (μm)	535.19 ± 29.56	2.14	5.92	0.40	0.995 (0.993–0.996)
AQD (mm)	2.86 ± 0.45	0.01	0.04	0.51	0.999 (0.998–0.999)
ACD (mm)	3.40 ± 0.45	0.02	0.04	0.46	0.999 (0.998–0.999)
Km (D)	43.70 ± 1.55	0.06	0.17	0.14	0.999 (0.998–0.999)
J_0_ (D)	−0.26 ± 0.42	0.04	0.12	-	0.989 (0.985–0.992)
J_45_ (D)	0.00 ± 0.21	0.04	0.12	-	0.957 (0.940–0.969)
CD (mm)	11.54 ± 0.39	0.04	0.12	0.38	0.987 (0.982–0.991)

*AL, axial length; CCT, central corneal thickness; AQD, aqueous depth; ACD, anterior chamber depth; Km, mean keratometry; CD, corneal diameter; SD, standard deviation; S_w_, within-subject standard deviation; TRT, test-retest repeatability (2.77 S_w_); CoV, within-subject coefficient of variation; ICC, intraclass correlation coefficient*.

### Measurement Agreements Between the Two Biometers

**Figure 1:**
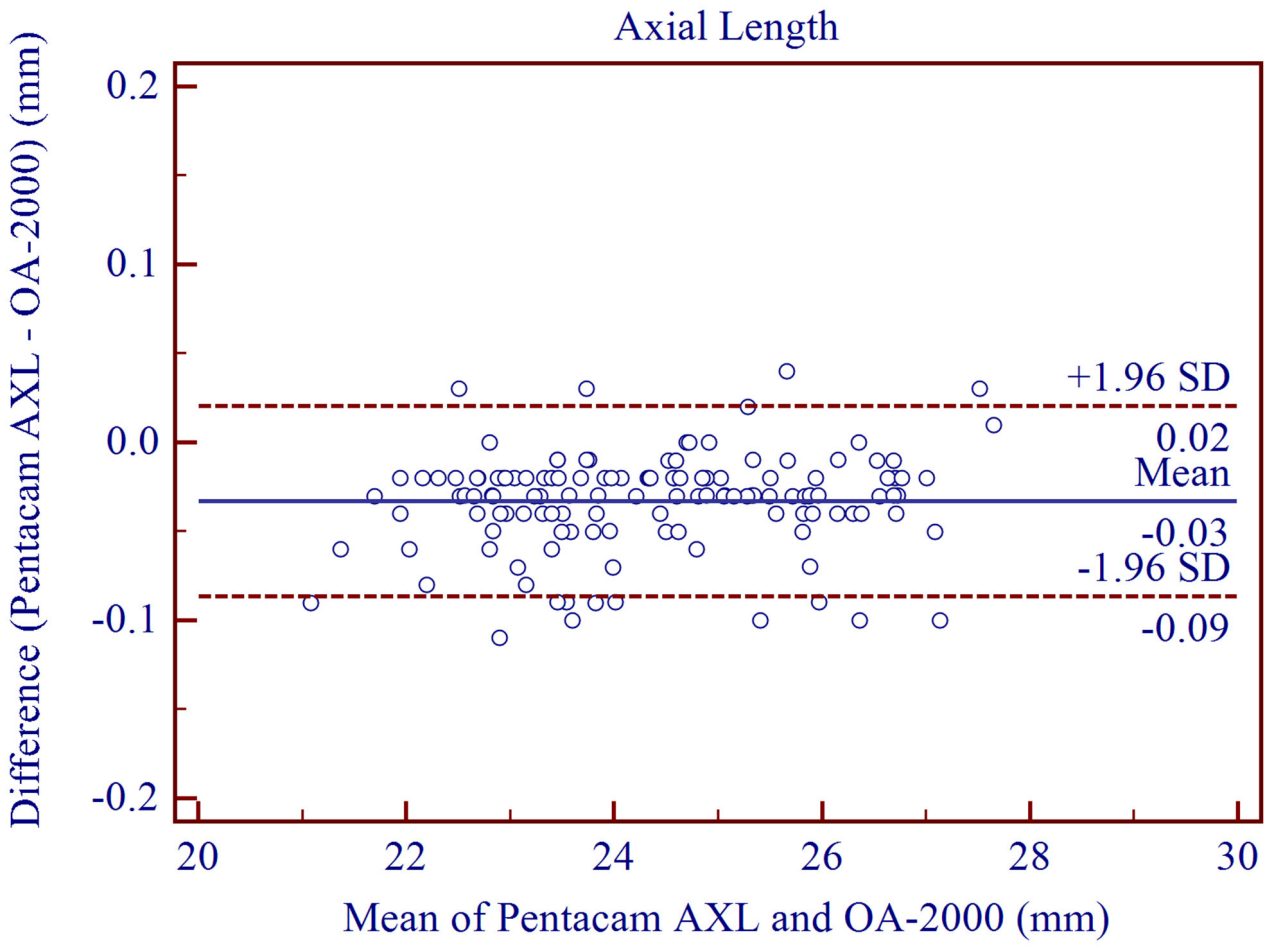
Bland-Altman plots showing agreement between the new Scheimpflug imager in combination with partial coherence interferometry biometer and the swept-source optical coherence tomography-based biometer for measuring axial length. Solid lines represent the bias between the two devices and dotted lines represent the 95% confidence interval for the difference.

**Figure 2:**
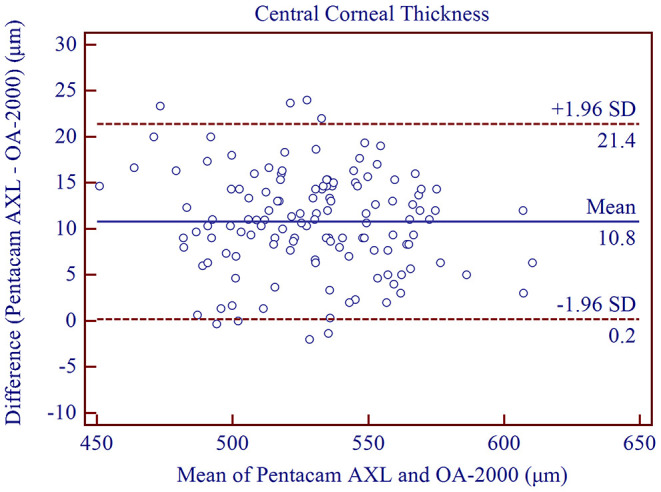
Bland-Altman plots showing agreement between the new Scheimpflug imager in combination with partial coherence interferometry biometer and the swept-source optical coherence tomography-based biometer for measuring central corneal thickness. Solid lines represent the bias between the two devices and dotted lines represent the 95% confidence interval for the difference.

**Figure 3:**
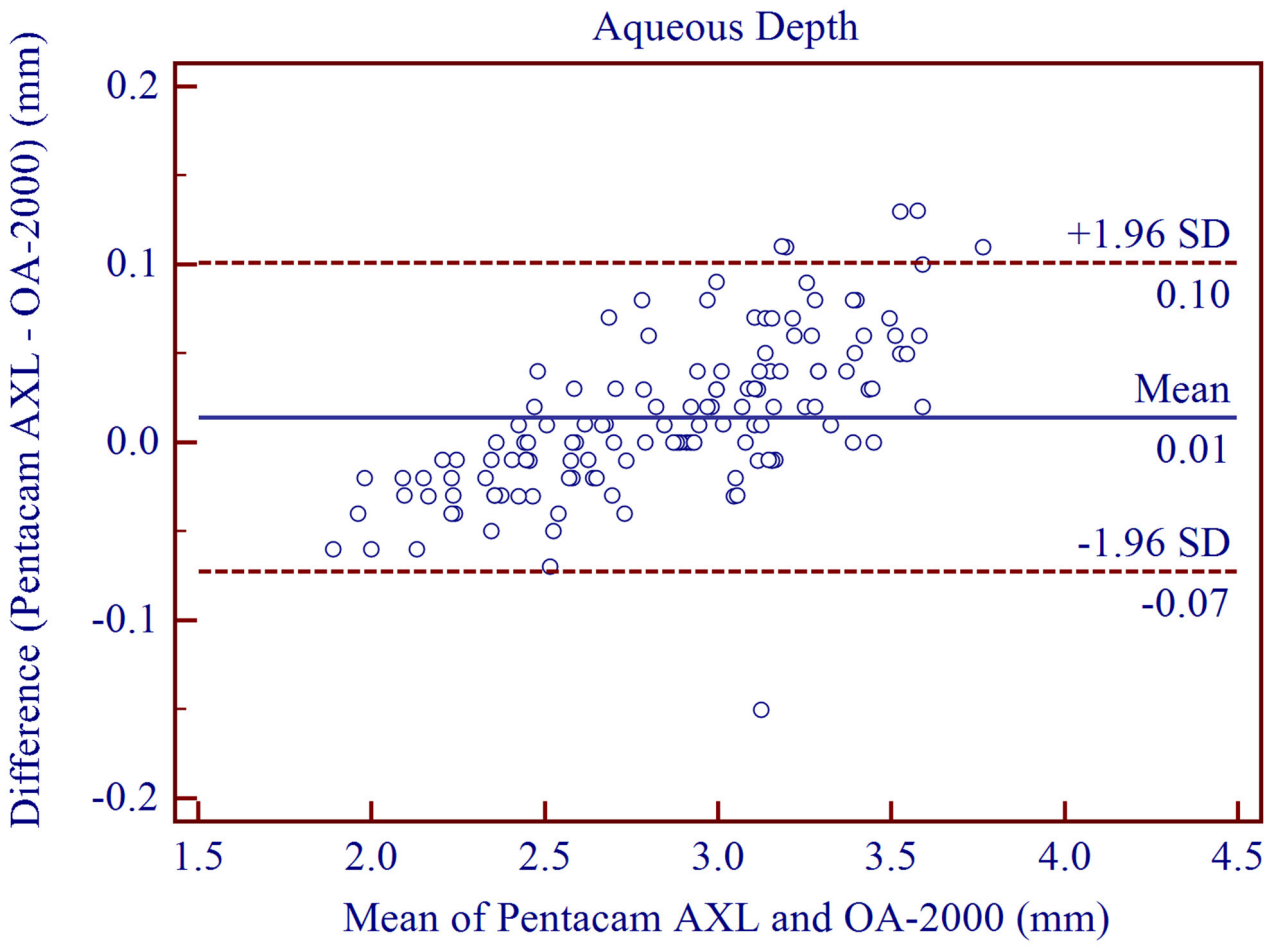
Bland-Altman plots showing the agreement between the new Scheimpflug imager in combination with partial coherence interferometry biometer and the swept-source optical coherence tomography-based biometer for measuring aqueous depth. Solid lines represent the bias between the two devices and dotted lines represent the 95% confidence interval for the difference.

**Figure 4:**
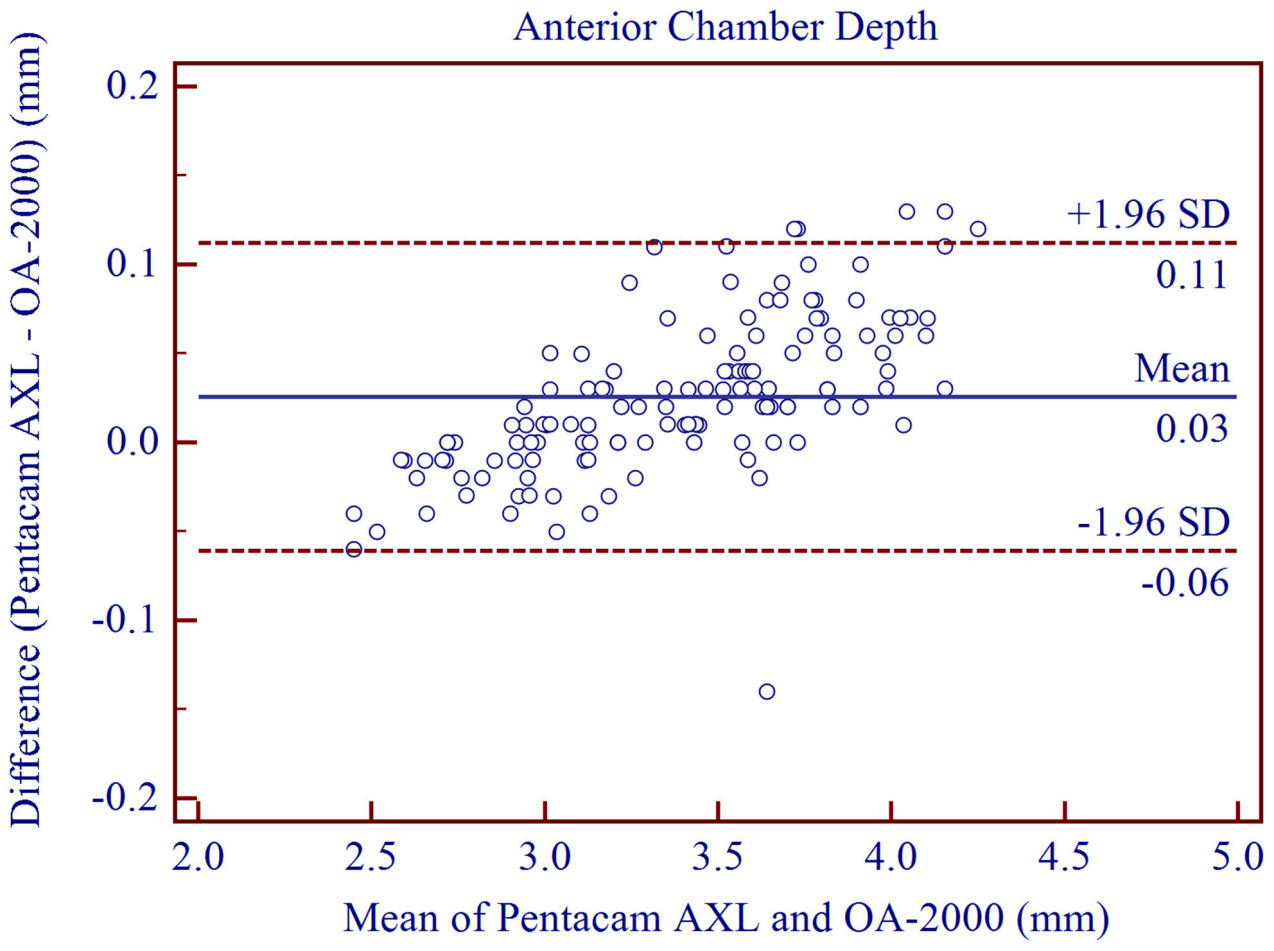
Bland-Altman plots showing the agreement between the new Scheimpflug imager in combination with partial coherence interferometry biometer and the swept-source optical coherence tomography-based biometer for measuring anterior chamber depth. Solid lines represent the bias between the two devices and dotted lines represent the 95% confidence interval for the difference.

**Figure 5:**
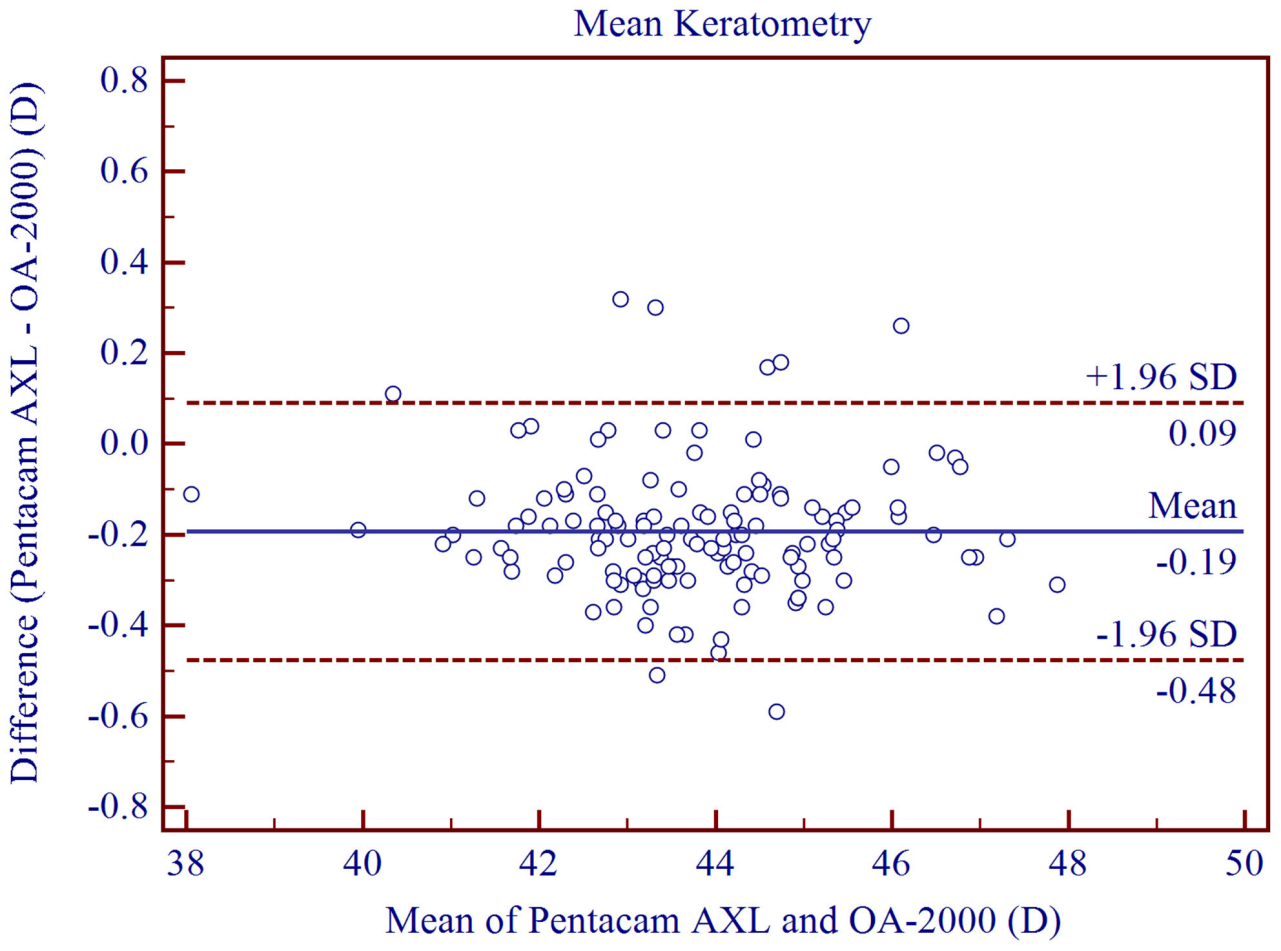
Bland-Altman plots showing the agreement between the new Scheimpflug imager in combination with partial coherence interferometry biometer and the swept-source optical coherence tomography-based biometer for measuring mean keratometry at 3.0 mm. Solid lines represent the bias between the two devices and dotted lines represent the 95% confidence interval for the difference.

The measurement agreement of the new biometer and the biometer with the combined SS-OCT and Placido-disk topography were excellent (Table 3). There were no statistically significant differences in J0 and J45 measurements (*P* > 0.05). For the other parameters, the differences were statistically significant (*P* < 0.001), but not clinically significant. The 95% LoA between the two biometers were narrow for most of the parameters, especially for AL, and were moderate for CCT (95% LoA, 0.18 to 21.41 μm). Bland-Altman plots for each parameter is illustrated in Figures 1–5 and Supplementary Figures 1–3.

**Table 3:** The mean difference, paired T-test, and 95% limits of agreement (LoA) for differences between the new Scheimpflug imager in combination with partial coherence interferometry biometer and the swept-source optical coherence tomography-based biometer.

**Device Pairings**	**Mean ±SD**	***P* Value**	**95% LoA**
AL (mm)	−0.03 ± 0.03	<0.001	−0.09 to 0.02
CCT (μm)	10.80 ± 5.41	<0.001	0.18 to 21.41
AQD (mm)	0.01 ± 0.04	<0.001	−0.07 to 0.10
ACD (mm)	0.03 ± 0.04	<0.001	−0.06 to 0.11
Km (D)	−0.19 ± 0.14	<0.001	−0.48 to 0.09
J_0_ (D)	0.01 ± 0.10	0.242	−0.18 to 0.20
J_45_ (D)	−0.01 ± 0.08	0.302	−0.17 to 0.15
CD (mm)	−0.20 ± 0.15	<0.001	−0.50 to 0.09

*AL, axial length; CCT, central corneal thickness; AQD, aqueous depth; ACD, anterior chamber depth; Km, mean keratometry; CD, corneal diameter; SD, standard deviation*.

## Discussion

The new ocular biometer, Pentacam AXL is considered an upgraded version of the Pentacam HR, which is based on Scheimpflug imaging and is commonly used to measure anterior segment parameters. With the corresponding optical biometer, PCI was included for AL measurement. However, only a few studies have investigated its measurement precision. In this study, all measurements using the Pentacam AXL demonstrated high precision. With respect to intra-observer repeatability for AL measurements, the ICC of the two observers were both 0.999 and was similar to the study performed by Saadettin et al. (19) (ICC = 0.995) and Güler et al. (20) (CoV = 0.11%, ICC = 0.998). To our knowledge, the current study was the first to assess interobserver reproducibility of this new biometer. Our results showed high reproducibility for AL measurements (CoV = 0.09%, ICC=1.000), and was similar to the study using another optical biometer performed by Yu et al. (21) (CoV = 0.09%, ICC = 1.000). Agreement between PCI and SS-OCT for AL was high, with a mean difference of only −0.03 ± 0.03 mm, which was not clinically significant. In addition, the 95% LoA was narrow (from −0.09 to 0.02 mm). The mean difference between PCI and SS-OCT in the study performed by Shajari et al. (5) was −0.026 mm with an average LoA of 0.11 mm. Srivannaboonet et. al. (22) compared SS-OCT (IOLMaster 700) and PCI (IOLMaster 500) for AL measurements of cataract eyes and observed high agreement (95% LoA, from −0.03 to 0.06 mm) with no statistically significant differences. Yang et al. (6) demonstrated that the SS-OCT biometer (IOLMaster 700) had a slightly longer AL compared to the PCI biometer (IOLMaster 500), with a mean difference of 0.060 ± 0.144 mm.

Excellent intra-observer CCT repeatability was observed in our study, with a CoV of 0.65% and ICC of 0.986. These results were similar to those reported by Paola et al. (23) (ICC = 0.982) and Crawford et al. (24) (CoV = 0.7%, ICC = 0.979). Previous studies had demonstrated good repeatability of Scheimpflug imaging (22, 25–29). Viswanathan et al. (30) evaluated the inter-observer reproducibility of the rotating Scheimpflug camera in normal and keratoconic eyes and observed excellent results for CCT measurement (ICC = 0.988). We demonstrated a similar ICC (0.986) and a lower CoV (0.65%) in our study using the Scheimpflug/PCI based biometer. Compared to measurements using the rotating Scheimpflug imager (Pentacam HR) with that of another optical biometer based on SS-OCT (IOLMaster 700), moderate CCT agreement was observed (31). The mean difference was 10.99 ± 7.57 μm and the 95% LoA was between −3.85 and 25.83 μm. Pelin et al. (32) demonstrated that the mean difference was −5.05 ±7.67 μm and the 95% LoA was between 9.8 and −19.9 μm. These results showed moderate agreement between Scheimpflug imaging and SS-OCT and were similar to our results.

We found that the optical biometer with the Scheimpflug camera had good intra-observer repeatability (TRT <0.07 mm, ICC > 0.997) and inter-observer reproducibility (TRT <0.04 mm, ICC > 0.998) for ACD measurements, and were in good agreement with results from previous studies. Wang et al. (2) assessed the intra-observer repeatability (TRT <0.08 mm, ICC > 0.986) and inter-observer reproducibility (TRT = 0.06 mm, ICC = 0.992) of the rotating Scheimpflug camera (Pentacam HR). They had excellent results for ACD measurements. The high precision of the dual-Scheimpflug-Placido (Galilei, Ziemer, Port, Switzerland) was also demonstrated by Mehmet et al. (33) and Altiparmak et al. (34). High agreement of ACD measurements between the biometer based on Scheimpflug imaging and the biometer based on SS-OCT was demonstrated in our study (95% LoA between −0.07 and 0.10 mm, mean difference: 0.03 ± 0.04 mm). These results are similar to previous studies comparing the Pentacam AXL and the IOLMaster 700 (5, 19).

Scheimpflug imaging has been demonstrated to have high precision in terms of repeatability and reproducibility for corneal curvature measurements (10, 30, 35, 36). Our data also showed good intra-observer repeatability (CoV <0.24%, ICC > 0.995) and inter-observer reproducibility (CoV <0.14%, ICC > 0.998) for Km measurements using the Pentacam AXL, and were similar to those reported by Ruiz-Mesa et al. (3). Mean K measurements showed a narrow range for 95% LoA (from −0.48 to 0.09 D), demonstrating good agreement between Scheimpflug imaging and Placido disk topography. A similar outcome was also demonstrated in our previous study (37) which compared Placido disk topographer (OphthaTOP, Hummel AG, Germany) and rotating Scheimpflug camera (Pentacam HR). The 95% LoA was between −0.45 and 0.09 D, and the mean difference was −0.18 ± 0.14 D. With respect to J0 and J45 measurements using the Scheimpflug based biometer, excellent intra-observer repeatability (ICCs: J0 > 0.972, J45 > 0.862, TRT: J0 = 0.17 D, J45 = 0.19 D) and inter-observer reproducibility (ICCs: J0 > 0.972, J45 > 0.862, TRT: J0 = 0.12 D, J45 = 0.12 D) were observed and were similar to those of previous studies (30, 38). Good agreement with the optical biometer and the Placido disk were observed with no significant differences and were similar to previous studies (P: J0 = 0.059, J45 = 0.133) (95% LoA: J0: −0.17 to 0.23 D, J45: −0.18 to 0.14 D) (37).

With regards to CD measurements, the Pentacam AXL had a high precision and moderate agreement with that of the SS-OCT biometer (the mean difference was −0.20 ± 0.15 mm, 95% LoA, −0.50 to 0.09 mm). The high precision was similar to those published by Lattimore et al. and Shajari et al. (39). However, Salouti et al. (40) showed poor agreement for CD measurements between the SS-OCT optical biometer (IOLMaster 700) and the same rotating Scheimpflug camera (95% LoA, −0.17 and 0.78 mm). These results suggest that the accuracy of CD measurements depends on the method that each device uses to define the limbus and the quality of the anterior segment images that are generated.

There were several limitations to this study. We only assessed the repeatability, reproducibility, and agreement between normal and cataract eyes and did not evaluate keratoconus, glaucomas, or post-refractive surgery patients. Additional studies are required to assess the repeatability, reproducibility, and agreement in patients with the above-mentioned conditions. Furthermore, we did not evaluate lens thickness measurements, as they require pupil dilation using the optical biometer based on Scheimpflug imaging and PCI.

In summary, the new optical biometer that utilized Scheimpflug imaging in combination with PCI provides repeatable and reproducible measurements of all parameters evaluated in this study. The measurement agreement between the two devices was high for most parameters. This suggests that the two biometers could be clinically interchangeable for the majority of measurements.

## Data Availability Statement

The original contributions presented in the study are included in the article/Supplementary Material, further inquiries can be directed to the corresponding authors.

## Ethics Statement

The studies involving human participants were reviewed and approved by the Research Review Board of Wenzhou Medical University. The patients/participants provided their written informed consent to participate in this study.

## Author Contributions

SC, JH, and RT: design and management. SC, QZ, and GS: conduct of the study and analysis. JY, YW, and RN: collection and preparation interpretation of data. SZ and XH: interpretation of data. SC, GS, JH, and RT: review. SC, QZ, GS, SZ, XH, JY, YW, RN, JH, and RT: approval of the manuscript. All authors contributed to the article and approved the submitted version.

## Funding

This work was supported by the Foundation of Wenzhou City Science and Technology Bureau (Y2020037) and Eye and ENT Hospital of Fudan University High-level Talents Program (2021318). The contribution of IRCCS G.B. Bietti Foundation was supported by Fondazione Roma and the Italian Ministry of Health. The sponsor or funding organization had no role in the design or conduct of this research.

## Conflict of Interest

The authors declare that the research was conducted in the absence of any commercial or financial relationships that could be construed as a potential conflict of interest.

## Publisher's Note

All claims expressed in this article are solely those of the authors and do not necessarily represent those of their affiliated organizations, or those of the publisher, the editors and the reviewers. Any product that may be evaluated in this article, or claim that may be made by its manufacturer, is not guaranteed or endorsed by the publisher.

## Supplementary Material

Supplementary Figure 1 Bland-Altman plots showing the agreement between the new Scheimpflug imager in combination with partial coherence interferometry biometer and the swept-source optical coherence tomography-based biometer for measuring J_0_. Solid lines represent the bias between the two devices and dotted lines represent the 95% confidence interval for the difference.

Supplementary Figure 2 Bland-Altman plots showing the agreement between the new Scheimpflug imager in combination with partial coherence interferometry biometer and the swept-source optical coherence tomography-based biometer for measuring J_45_. Solid lines represent the bias between the two devices and dotted lines represent the 95% confidence interval for the difference.

Supplementary Figure 3 Bland-Altman plots showing the agreement between the new Scheimpflug imager in combination with partial coherence interferometry biometer and the swept-source optical coherence tomography-based biometer for measuring corneal diameter. Solid lines represent the bias between the two devices and dotted lines represent the 95% confidence interval for the di?erence.
