# Supplementary figures and images for: Comparison of a New Optical Biometer That Combines Scheimpflug Imaging With Partial Coherence Interferometry With That of an Optical Biometer Based on Swept-Source Optical Coherence Tomography and Placido-Disk Topography
Source: Front Med (Lausanne). 2022 Feb 10;8:814519. doi: 10.3389/fmed.2021.814519 (PMC8866319; doi:10.3389/fmed.2021.814519)

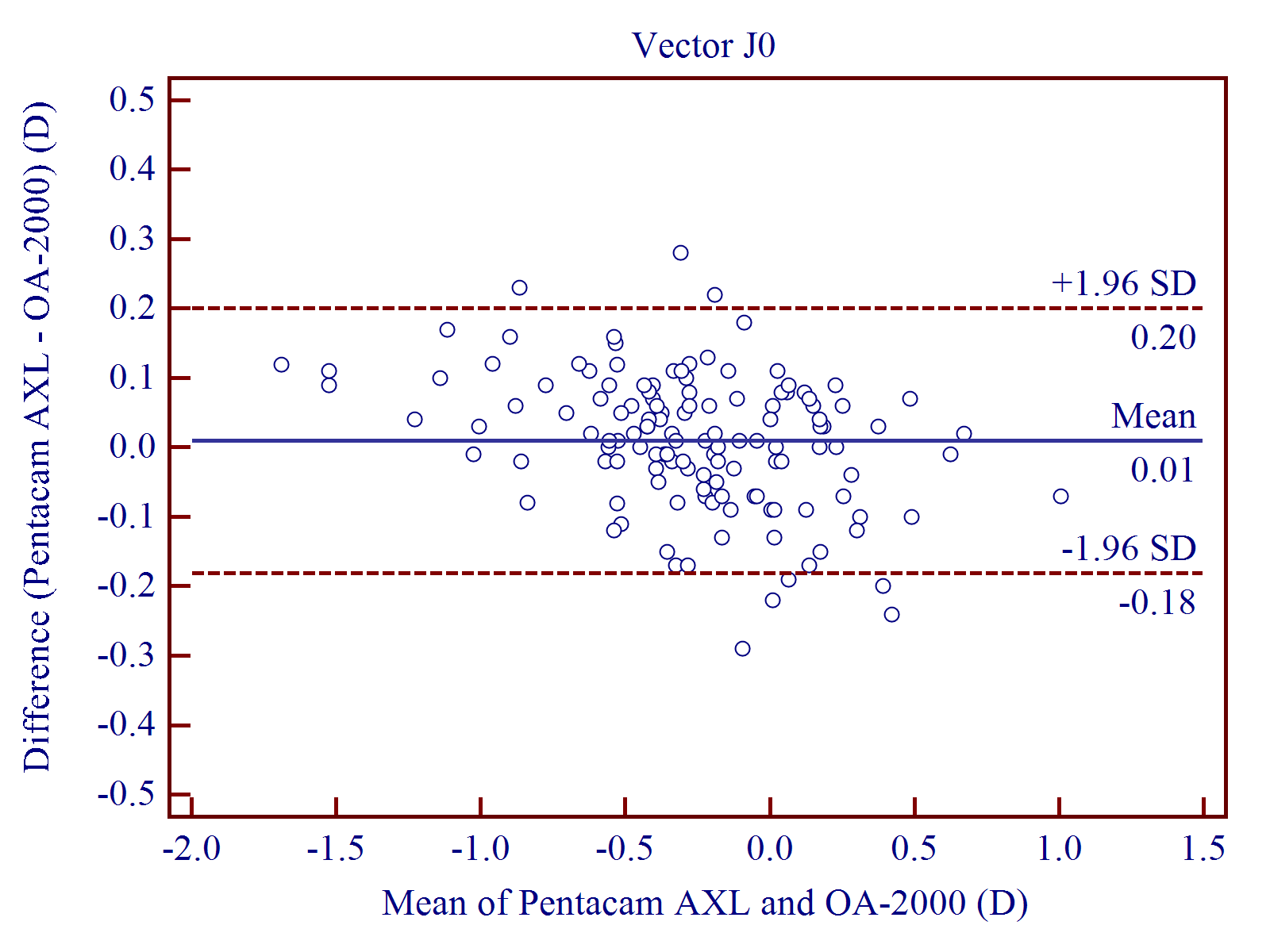

Supplement: Supplementary Figure 1 — Bland-Altman plots showing the agreement between the new Scheimpflug imager in combination with partial coherence interferometry biometer and the swept-source optical coherence tomography-based biometer for measuring J0. Solid lines represent the bias between the two devices and dotted lines represent the 95% confidence interval for the difference. [file Image_1.TIF]

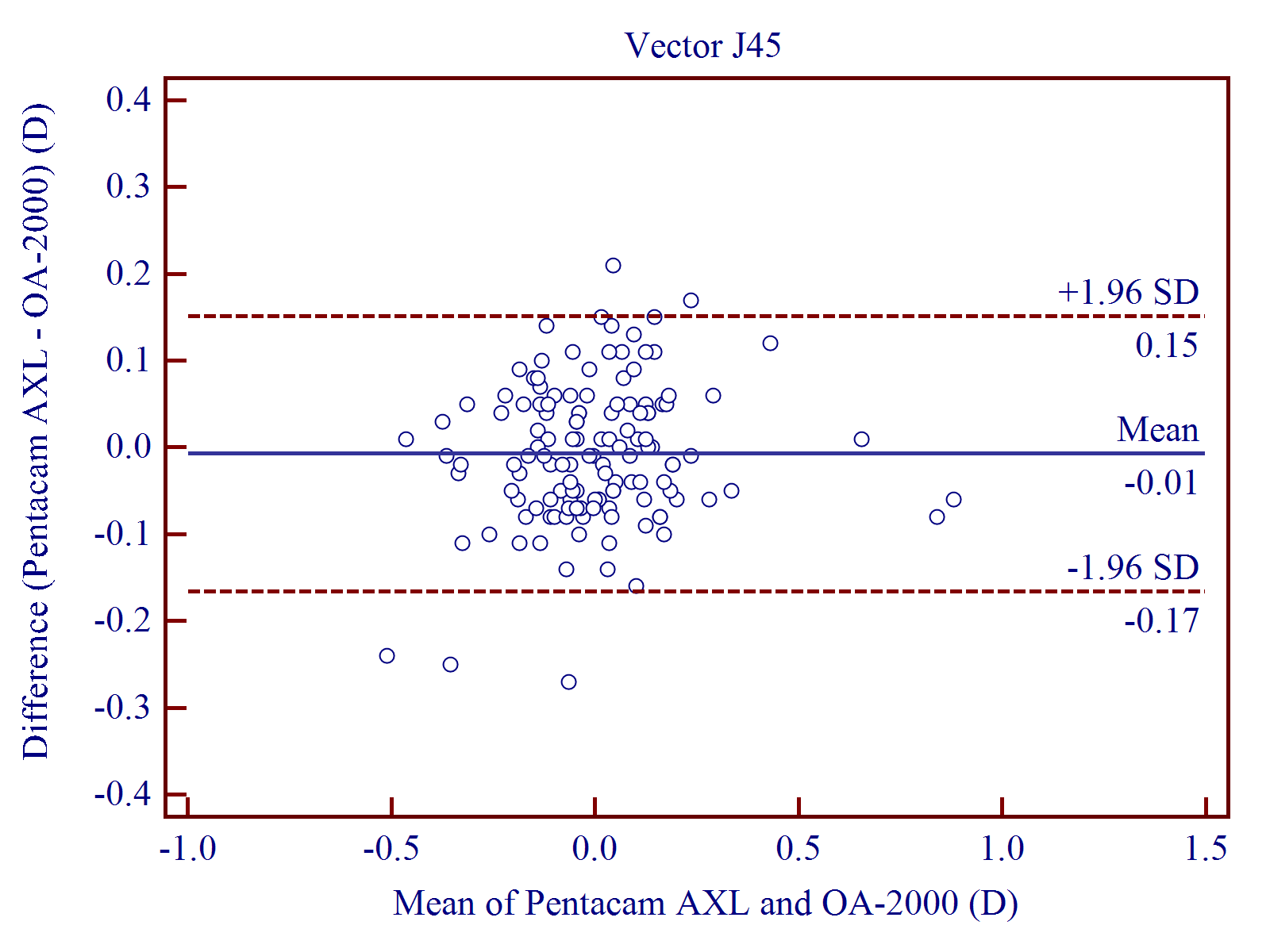

Supplement: Supplementary Figure 2 — Bland-Altman plots showing the agreement between the new Scheimpflug imager in combination with partial coherence interferometry biometer and the swept-source optical coherence tomography-based biometer for measuring J45. Solid lines represent the bias between the two devices and dotted lines represent the 95% confidence interval for the difference. [file Image_2.TIF]

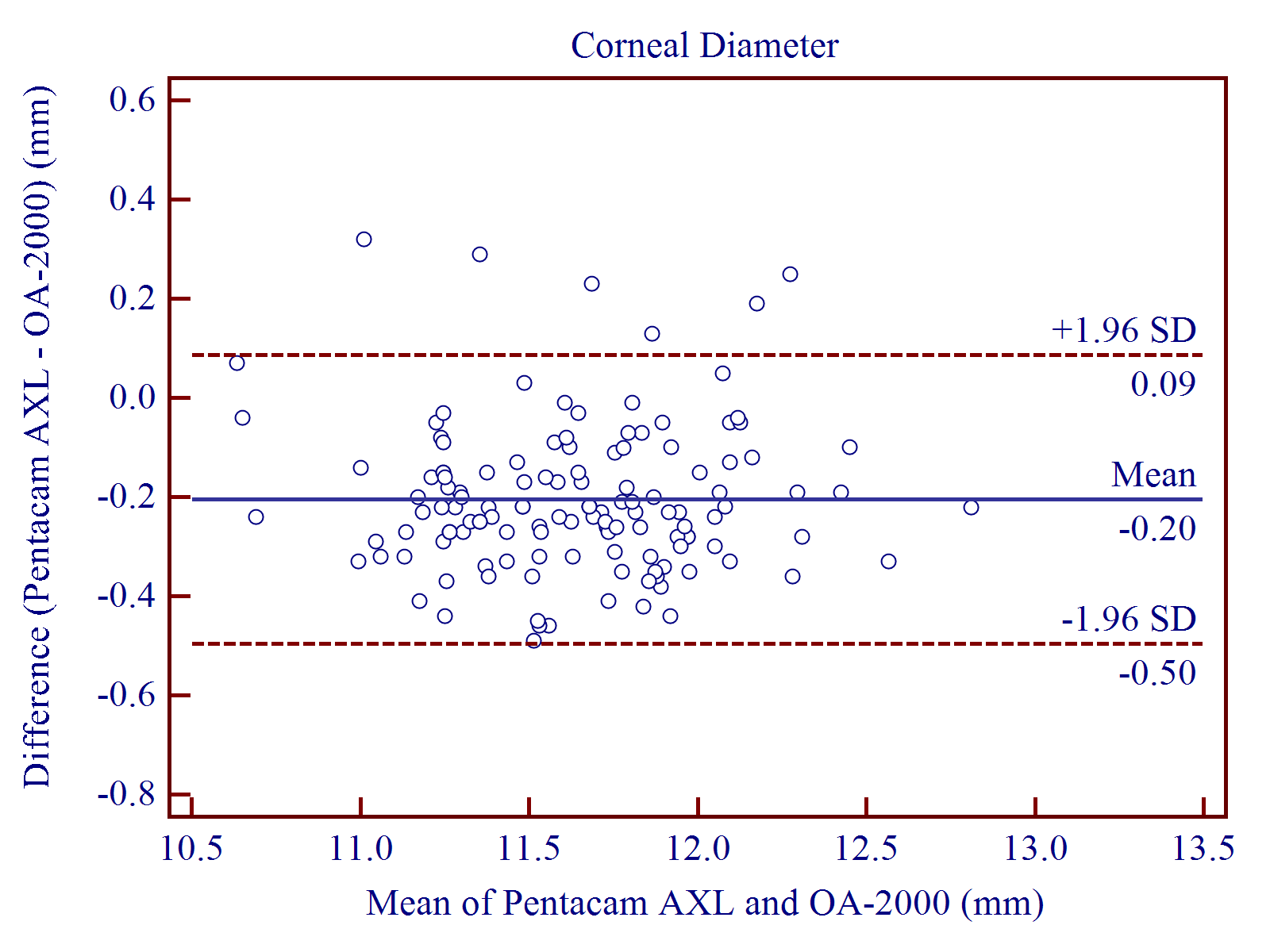

Supplement: Supplementary Figure 3 — Bland-Altman plots showing the agreement between the new Scheimpflug imager in combination with partial coherence interferometry biometer and the swept-source optical coherence tomography-based biometer for measuring corneal diameter. Solid lines represent the bias between the two devices and dotted lines represent the 95% confidence interval for the di?erence. [file Image_3.TIF]
